# Are psychosocial work factors and work-home interference associated with time to first full return-to-work after sick leave due to common mental disorders?

**DOI:** 10.1007/s00420-023-01970-z

**Published:** 2023-03-25

**Authors:** Lisa Holmlund, Ute Bültmann, Gunnar Bergström, Anna Warnqvist, Elisabeth Björk Brämberg

**Affiliations:** 1 Institute of Environmental Medicine, Unit of Intervention and Implementation Research for Worker Health, Karolinska Institutet, P.O.Box 210, 171 77 Stockholm, Sweden; 2 Department of Health Sciences, Community and Occupational Medicine, University of Groningen, University Medical Center Groningen, Groningen, The Netherlands; 3 Department of Occupational Health Sciences and Psychology, Faculty of Health and Occupational Studies, University of Gävle, Gävle, Sweden; 4 Institute of Environmental Medicine, Division of Biostatistics, Karolinska Institutet, Stockholm, Sweden

**Keywords:** Determinants, Mental disorders, Return to work, Sickness absence, Survival analysis, Work factors

## Abstract

**Objective:**

To (1) examine the time to first full return-to-work (RTW), and (2) investigate whether psychosocial work factors and work-home interference are associated with time to first full RTW after sick leave due to common mental disorders (CMDs).

**Methods:**

The cohort study comprised 162 employees on sick leave due to CMDs participating in a two-armed cluster-randomised controlled trial in Sweden. Baseline data consisted of a web-based questionnaire and follow-up data of repeated text messages every fourth week for 12 months. The time to first full RTW was estimated using the Kaplan–Meier Estimator. Parametric Weibull survival models with interval-censored outcomes were used to determine associations between psychosocial work factors and work-home interference with time to first full RTW. In a post hoc analysis, time-interval differences in associations for 0– ≤ 6- versus > 6–12 months were tested.

**Results:**

During the 12-month follow-up, *n* = 131 (80.9%) reported a first full RTW. The median time to this RTW was 16 weeks (95% CI 12; 20). High psychological job demands, high emotional job demands, high work-to-home interference (WHI), and low social job support were independently associated with a longer time to first full RTW. Time-interval differences were found for job control and emotional job demands.

**Conclusions:**

Psychosocial work demands and WHI are associated with a longer time to RTW after sick leave due to CMDs. Work organisations and rehabilitation practices should include accommodations for high psychological and emotional job demands during RTW, as well as pay attention to the risk of spill-over of high job demands into employees’ private lives.

**Supplementary Information:**

The online version contains supplementary material available at 10.1007/s00420-023-01970-z.

## Introduction

Return-to-work (RTW) is conceptually framed as a complex process involving the individual and the environment (Nielsen et al. 2018; Young et al. 2005). As sick leave due to common mental disorders (CMDs) impacts the individual, the workplace, and society at large (OECD 2018) knowledge that can inform strategies for a sustainable RTW is important for the individual, employers, health care professionals, and policymakers. Earlier theoretical models (Demerouti et al. 2001; Karasek and Theorell 1990) and research (Aronsson et al. 2017; de Vries et al. 2018; Harvey et al. 2017; Duchaine et al. 2020) have identified psychosocial demands at work, such as high psychological job demands, low job control, and low social job support, as associated with the development of CMDs and resulting sick leave. Similarly, work-home interference, i.e. work-to-home interference (WHI) and home-to-work interference (HWI) have been associated with CMDs and sick leave (Blom et al. 2014; Svedberg et al. 2018). To date, less is known about the impact of psychosocial work factors, work-home interference, and the time to RTW (cf. de Vries et al. 2018; Joosen et al. 2021; Nybergh et al. 2020). The employer has a central role to accommodate the employee in RTW after sick leave due to CMD (Gensby et al 2019; Corbiere et al. 2020; Joosen et al. 2021; Nybergh et al. 2020). Understanding associations between RTW, psychosocial work factors, and work-home interference has the potential to guide organisational measures for RTW.

To date, a few studies have found associations between high psychological job demands (Ekberg et al. 2015; Flach et al. 2012; Netterstrøm et al. 2015) and low job control (Flach et al. 2012; Netterstrøm et al. 2015) and RTW after sick leave due to CMDs. However, in a recent scoping review, de Vries et al. (2018) found the evidence for many factors influencing RTW, including psychological job demands, to be insufficient. Similarly, the evidence for the association between low social job support in the workplace and RTW is inconclusive (Flach et al. 2012; Netterstrøm et al. 2015; Sikora et al. 2022). A good balance in everyday life is perceived as essential for a ‘successful’ RTW, while interference between demands at work and in private life has been identified as a barrier (Hees et al. 2012; Joosen et al. 2021; Nybergh et al. 2020). Because of the gendered structure of demands in private life, the negative effect of domestic strain on sick leave and RTW is greater among women (Holmlund et al. 2022; Nybergh et al. 2020; Östlund et al. 2004). To date, there is limited evidence of an association between WHI and HWI and time to RTW. Only recently, Sikora et al. (2022) found that employees receiving in-patient treatment for their CMDs and reporting work-home interference as associated with a longer time to first and full RTW.

When investigating RTW it is important to consider the administrative setting (Krause et al. 2001a; Ståhl et al. 2018). The associations between work factors, work-home interference, and RTW can differ between countries or between phases of the RTW process depending on cultural and legal systems (Krause et al. 2001a, b). In Sweden, the risks associated with work factors and work-home interference for both genders are interesting because labour market participation is nearly equal for men and women. Moreover, factors associated with time to RTW are interesting because of policies implemented in Sweden to provide efficient RTW. Examples of this are employers’ responsibility to design an RTW plan within 30 days of sick leave (if the employee is expected to be on sick leave for at least 60 days) and time intervals for eligibility for sick leave. In Sweden, after > 6 months of sick leave, an assessment of eligibility for any job in the entire labour market is carried out, whereas at 0– ≤ 6 months an assessment is carried out of the person’s ability to take on work at their present workplace (exceptions are made in cases of serious illness). A better understanding of work factors and work-home interference and time to RTW can lead to improved RTW practices. Moreover, a phase-specific analysis may show time-dependent influences of factors in specific settings (Krause et al. 2001a, b).

This study aimed to (1) examine the time to first full RTW, and (2) investigate whether psychosocial work factors and work-home interference are associated with time to first full RTW after sick leave due to CMDs. A post hoc analysis investigated whether the associations differ between employees returning to work between 0 and ≤ 6- and between > 6 and 12 months.

## Methods

### Study design

The study was conducted in Sweden’s Västra Götaland Region. It used baseline and follow-up data from a two-armed cluster-randomised controlled trial (RCT) evaluating a problem-solving intervention for reducing sick leave among employees sick-listed due to CMDs (reg. NCT3346395) (Björk Brämberg et al. 2018). The original study sample consisted of 197 individuals clustered by primary care centres (PCCs). The reporting of this study follows the STROBE checklist and includes a cohort of 162 individuals followed up for 12 months.

### Participants and procedures

Participants in the cluster-RCT were recruited between February 2018 and February 2020 from primary care centres (PCCs) in Sweden’s Västra Götaland Region (Björk Brämberg et al. 2018). Inclusion criteria were (1) employees aged 18–59; (2) on sick leave (minimum 2 weeks, maximum 12 weeks) diagnosed by a physician with mild to moderate depression, anxiety, or adjustment disorder (F 32, F 41, F 43) as the primary reason for sick leave; (3) participants accepted employer involvement and understood written and spoken Swedish. Exclusion criteria were (1) severe depression; (2) other severe mental disorders (psychotic or bipolar disorders or referral to a psychiatrist); (3) pregnancy; (4) somatic complaints or disorders that affect workability. 1511 individuals were eligible for the study and received written information.

A web-based questionnaire for baseline data was sent at inclusion and was responded to by 93.4%. Follow-up data were collected by text messages that were sent every fourth week after the baseline questionnaire for 12 months. Baseline data and follow-up data were self-reported and the data collection was monitored by research assistants blinded for group assignment. For the present study, *n* = 18 were excluded from the original sample (*n* = 197) because they had returned to work at baseline; *n* = 13 did not respond to the baseline questionnaire, while *n* = 4 did not respond to the follow-up text messages. A total of 1944 text messages were sent out during follow-up (1 question × 12 months × 162 participants) with a response rate of 90.2%.

### Measures

#### Time to first full RTW

Time to first full RTW was defined as the time to return to ordinary working hours (i.e. the hours they had worked before sick leave) for an uninterrupted period of 4 weeks. Time to first full RTW was assessed by a web-based questionnaire starting at baseline: ‘During the last 4 weeks, have you worked your ordinary hours for an uninterrupted period of at least 4 weeks’ (answer 1 = yes, 2 = no). During the 12-month follow-up, the following question was sent by text message every fourth week: “During the last 8 weeks, have you worked your ordinary working hours for an uninterrupted period of at least 4 weeks’ (1 = yes, 2 = no).

#### Psychosocial work factors and work-home interference

Job demands and resources were measured using subscales or single items of validated instruments (Berthelsen et al. 2020; Sanne et al. 2005; Wännström et al. 2009). Psychological job demands, job control, and social job support were measured on a four-point Likert scale using the Swedish Demand–Control–Support Questionnaire (DSCQ). The DSCQ is a shortened and modified version of the Job Content Questionnaire and includes the scales (1) psychological job demands (five items); (2) decision latitude (six items); (3) social support (six items) (Karasek et al. 1998; Sanne et al. 2005). Higher scores indicated higher psychological job demands, job control, and social job support. Emotional job demands (three items) were measured on a five-point Likert scale using the Copenhagen Psychosocial Questionnaire III (COPSOQ III) (Berthelsen et al. 2020). Fair leadership (1 item) and work-home interference (2 items, i.e., WHI and HWI) were measured on a five-point Likert scale using the General Nordic Questionnaire for Psychological and Social Factors at Work (QPS-Nordic) (Wännström et al. 2009). The following questions were asked: (Fair leadership) ‘Does your nearest superior treat workers fairly and equally?’; (WHI) ‘Do the demands of your work interfere with your home and family life?’; and (HWI) ‘Do the demands of your family or spouse/ partner interfere with your work-related activities?’ For the DSCQ the mean was scaled with the number of questions in the dimension. The COPSOQ dimension was calculated following Berthelsen (2020) by taking the mean of the questions and scaling it by 25. If > 50% of the items on a scale were not answered, the scale score was not calculated. For further details about the scales, see supplementary file 1.

#### Socio-demographic, employment, and clinical factors

Employee characteristics and employment information were collected by a web-based questionnaire at baseline. Sociodemographic characteristics were age, gender, country of origin, cohabitation status, children living at home (under the age of 16 years), household responsibilities, and level of education (primary/secondary, higher education/university). Employment information was contract type, sector (municipality/county/state, private, other), type of work (mentally demanding, physically demanding, both), ordinary working hours (full-time, part-time), and work tenure in years (≤ 2, 3–5, ≥ 6). Sick leave information was full-time (100%) or part-time (25/50/75%) sick leave of ordinary working hours. Baseline diagnosis was collected from the Swedish Social Insurance Agency’s (SSIA) register Micro Data for the Analysis of Social Insurance register (MiDAS).

Covariates were collected from the baseline variables and included the variables age, level of education, sick leave from ordinary working hours, and randomisation (intervention, control).

### Statistical analysis

Descriptive statistics were calculated for all participants and the proportion of participants with and without an event of first full RTW. The time to first full RTW during the 12-month follow-up was estimated using the Kaplan–Meier estimator. The percentages of employees reporting RTW for four consecutive weeks at each specific time point (every fourth week) were illustrated using a bar graph.

To examine whether psychosocial work factors and work-home interference are associated with first full RTW, a parametric Weibull survival regression model with interval-censored outcome was conducted. Interval censoring was used because the exact date of RTW was not known. If the preceding values to the first full RTW were missing, the lower limit of the censoring interval was extended to match the period with no information. If a missing value was preceded and followed by a reported non-working period, the subject was considered not to have returned to work. Observations were censored if lost to follow-up or at the end of the study (Finkelstein 1986; Sun 2006). The effect of each covariate of interest was estimated using an unadjusted model and using an adjusted model including age and education; sick leave at baseline; and randomisation group. Assumptions and model fit were checked using Cox-Snell residuals. To take into consideration the intra-cluster correlation inherent to the specific care centers, a sensitivity analysis was done. This analysis was adjusted for the care center. Despite the lower power, the conclusions of the sensitivity analysis were in line with the primary analysis.

A post hoc analysis was conducted using a multivariate model dividing the data into two-time intervals: 0– ≤ 6- versus > 6–12 months. Weibull survival regression was first used to investigate the association between each variable of interest and the first full RTW during the two-time intervals separately. Thereafter, we used the interaction term between the time interval and the variable of interest to investigate whether the associations between the different variables and time to first full RTW differed between the two-time intervals. All analyses were conducted in Stata, version 15 (StataCorp. 2017. *Stata Statistical Software: Release 15*. College Station, TX: StataCorp LLC.). A *p*-value smaller than 0.05 was considered statistically significant.

## Results

### Sample characteristics

Of the 162 participants, 84.6% were women and the mean age was 42.5 years (SD 9.9). Table 1 shows the baseline characteristics of all participants. Between 0– ≤ 6- and > 6–12 months, 18.5% (*n* = 30) and 14.2% (*n* = 23), respectively, of the participants changed employers.

**Table 1:** Employee characteristics at baseline, participants with a first event of RTW and no event of RTW at 12-month follow-up

	All (*n* = 162)	RTW (*n* = 131)	No RTW (*n* = 31)
Age, years, m (SD)	42.5 (9.9)	42.7 (9.9)	41.4 (9.7)
Female, *n* (%)	137 (84.6)	111 (84.7)	26 (83.9)
Diagnosis^a^, *n* (%)			
Depressive disorder (F 32)	39 (24.1)	30 (22.9)	9 (29.0)
Anxiety disorder (F 41)	36 (22.2)	27 (20.6)	9 (29.0)
Adjustment disorder (F 43)	87 (53.7)	74 (56.5)	13 (41.9)
Born in Sweden, *n* (%)	156 (96.3)	125 (95.4)	31 (100.0)
Living with a partner, *n* (%)	122 (75.3)	100 (76.3)	22 (71.0)
Children living at home^b^, *n* (%)	95 (58.6)	78 (60.0)	17 (54.8)
Main household responsibility, *n* (%)			
Myself	89 (54.9)	72 (55)	17 (54.8)
Someone else	8 (4.9)	7 (5.3)	1 (3.2)
Equal share	65 (40.1)	52 (39.7)	13 (41.9)
Education level, *n* (%)			
Primary/secondary education	86 (53.1)	68 (51.9)	18 (58.1)
Higher education/university	76 (46.9)	63 (48.1)	13 (41.9)
Permanent employment, *n* (%)	151 (93.2)	122 (93.1)	29 (96.7)
Employer^c^, *n* (%)			
Municipality, county, state^d^	93 (57.8)	78 (59.5)	15 (50.0)
Private business	62 (38.5)	48 (36.6)	14 (46.7)
Other	6 (3.7)	5 (3.8)	1 (3.3)
Type of employment^c^, *n* (%)			
Mentally demanding	84 (52.2)	69 (52.7)	15 (50.0)
Physically demanding	7 (4.4)	7 (5.3)	–
Both mentally and physically demanding	70 (43.5)	55 (42.0)	15 (50.0)
Ordinary working hours^c^, *n* (%)			
Full-time (40 h/week)	114 (70.8)	93 (71)	21 (70.0)
Part-time (< 40 h/week)	47 (29.2)	38 (29)	9 (30.0)
Work tenure^c^, years, *n* (%)			
≤ 2	64 (39.8)	55 (41.0)	9 (30.0)
3–5	38 (23.6)	31 (23.7)	7 (23.3)
≥ 6	59 (36.7)	45 (34.4)	14 (46.7)
Sick leave^e^, *n* (%)			
Full-time	69 (50.0)	50 (45.0)	19 (70.4)
Part-time	69 (50.0)	61 (54.9)	8 (29.6)

*RTW* return-to-work, % Valid percent

^a^Data on diagnoses obtained from the Swedish Social Insurance Agency’s (SSIA) register Micro Data for the Analysis of Social Insurance register (MiDAS). If diagnoses were not registered in MiDAS, data were obtained from medical records

^b^Data missing for *n* = 1, group RTW

^c^Data missing for *n* = 1, group no RTW

^d^Employed by the state, *n* = 8

^e^Data missing for *n* = 24, RTW *n* = 20, No RTW *n* = 4

### Time to first full RTW

**Fig. 1:**
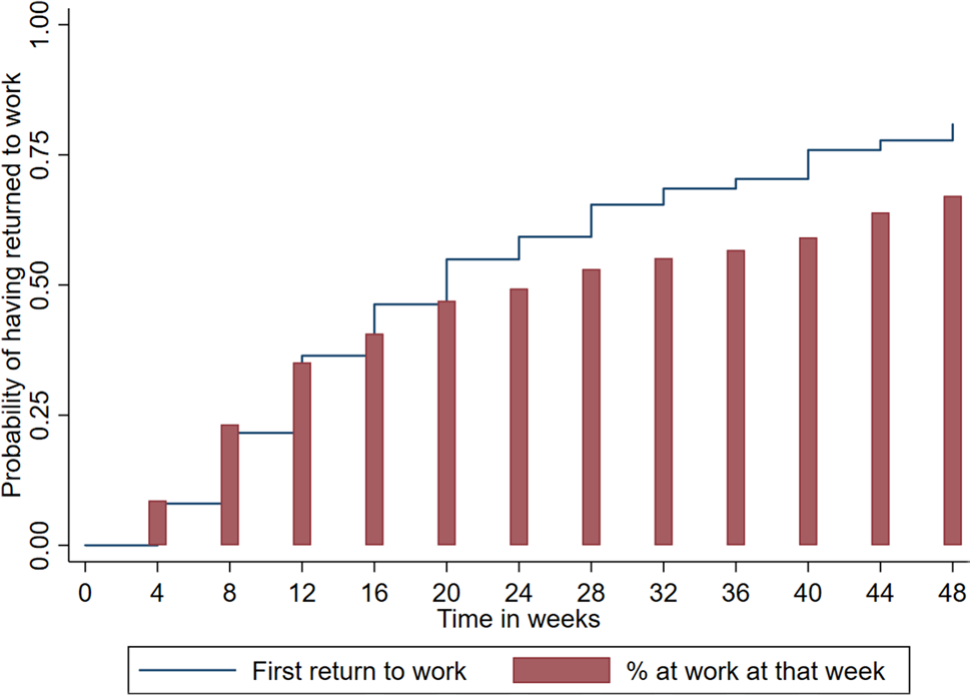
Kaplan–Meier estimate of the probability of the first full return-to-work (% of the sample) during each 4-week period of the 12-month follow-up; and the proportion of the sample (%) reporting that they have worked their ordinary working hours for 4 weeks for each time period

During the 12-month follow-up, *n* = 131 (80.9%) reported a first full RTW. The median time to first full RTW was 16 weeks (95% CI 12; 20). Figure 1 illustrates the time to first full RTW during the follow-up period and the proportion of the sample at work at each time point. A total of *n* = 96 (59.3%) reported their first full RTW between 0 and ≤ 6 months, and *n* = 35 (21.6%) between > 6 and 12 months. At the 12-month follow-up, 67% of the participants reported a period of uninterrupted work for 4 weeks.

### Factors associated with time to first full RTW

**Fig. 2:**
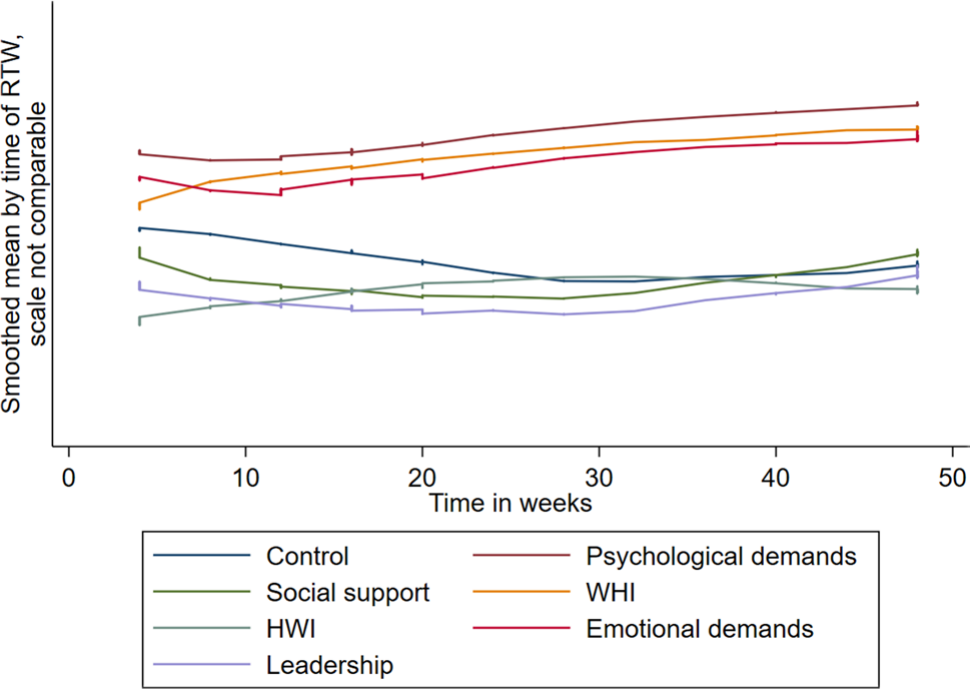
Smoothed mean by the time of RTW for psychosocial work factors and work-home interference. Each variable is standardised to have a scale between 0 and 1. Time was measured for all subjects as time from entering the study to RTW or lost to follow-up. Increased values on the Y-axis show lower control, lower social control, higher home-to-work interference (HWI), unfair leadership, higher psychological demands, higher work-to-home interference (WHI), and higher emotional demands

Figure 2 illustrates that over the 12-month follow-up, high psychological job demands and high WHI were consistently associated with a longer time to first full RTW. For other variables, such as low job control, the association with time to first full RTW changed over time. For example, during the first six months, low job control was associated with a shorter time to RTW, while the association during the last six months was the opposite. In Table 2, high psychological job demands (adj HR 0.95 95% CI 0.90–1.00), high emotional job demands (adj HR 0.99 95% CI 0.98–1.00), high WHI (adj HR 0.63 95% CI 0.43–0.93), and low social job support (adj HR 0.93 95% CI 0.88–0.99) were all independently associated with a longer time to first full RTW. See supplementary file 2 for descriptive information about the variables for psychosocial work factors and work-home interference, and supplementary file 3 for the effect of each covariate of interest in unadjusted and adjusted models.

**Table 2:** Hazard ratio of first full RTW during the 12-month follow-up, unadjusted and adjusted models

	No. (%) (*n* = 162)	0–12 months, Unadjusted Model		0–12 months, Adjusted Model^1^	
		HR (95% CI)	*p*	HR (95% CI)	*p*
Psychological job demands	–	0.94 (0.89–0.99)	0.02	0.95 (0.90–1.00)	0.05
Emotional job demands	–	0.99 (0.99–1.00)	0.07	0.99 (0.98–1.00)	0.04
Job control	–	1.03 (0.96–1.10)	0.46	1.04 (0.96–1.12)	0.33
Social job support	–	0.96 (0.92–1.02)	0.17	0.93 (0.88–0.99)	0.02
Fair leadership					
Ref. Fair	124 (76.5)	1		1	
Unfair	35 (21.6)	0.92 (0.60–1.42)	0.72	0.94 (0.58–1.53)	0.80
WHI					
Ref. Low	69 (42.6)	1		1	
High	93 (57.4)	0.78 (0.55–1.11)	0.17	0.63 (0.43–0.93)	0.02
HWI					
Ref. Low	123 (75.9)	1		1	
High	39 (24.1)	0.93 (0.62–1.39)	0.71	0.79 (0.50–1.25)	0.32

*HR* Hazard ratio, *WHI* work-to-home interference, *HWI* home-to-work interference, *RTW* return-to-work

HR < 1 indicates an increased risk of prolonged RTW (for the continuous variables), with higher psychological demands, higher emotional job demands, lower job control and lower social job support

Binary variables are dichotomized into fair/unfair (unfair = rather seldom and very seldom or never) and high/low WHI and HWI (high = rather often and very often or always)

^1^Adjusted by age and education, sick leave at baseline, and randomisation

*p* < 0.05

### Post hoc analysis

The post hoc interaction analysis showed that the associations for low job control (*p* < 0.01) and high emotional job demands (*p* = 0.04) and time to first full RTW significantly differed between 0– ≤ 6- and > 6–12 months. Between 0 and < 6 months, low job control was associated with a shorter time to first full RTW (HR 1.09 95% CI 1.00–1.18), while high emotional job demands were associated with a longer time to first full RTW (HR 0.99 95% CI 0.98–1.00). Between > 6 and 12 months, low job control was associated with a longer time to first full RTW (HR 0.85 95% CI 0.73–0.99), while high emotional job demands were not associated with a longer time to RTW (HR 1.00 95% CI 0.99–1.01). Results for all variables are presented in Supplementary file 4.

## Discussion

This study showed that 80.9% of the participants achieved a first full RTW during the 12-month follow-up, with the majority (59.3%) within the first six months. High psychological and emotional job demands, high WHI, and low social job support at baseline were independently associated with a longer time to first full RTW. Significant differences between time intervals (0– ≤ 6- and > 6–12 months) were found in a post hoc analysis of the associations between low job control and high emotional job demands, and time to first full RTW. Low job control was associated with a shorter time to first full RTW between 0 and ≤ 6 months and with a longer time to first full RTW between > 6 and 12 months.

A direct comparison across studies of time to RTW after CMDs is difficult due to differences in administrative settings and design. Our results are in line with a recent Swedish RCT conducted in occupational health services among employees absent from work due to CMDs. Here, 82% had returned to their ordinary working hours during a 12-month follow-up (Keus van de Poll et al. 2020). A German study of patients receiving in-patient care for CMDs found that 93.7% returned to work after 18 months of follow-up (Sikora et al. 2022). In a Danish study of employees on sick leave with CMDs enrolled from PCCs, using an outcome of full-time work at 12 months, 67% returned to full-time work after 1 year (Netterstrøm et al. 2015). The Danish results correlate with our last point estimate. In our study, as in the study by Keus van de Poll et al. (2020) and Sikora et al. (2022), the outcome was a first event of working ordinary working hours for an uninterrupted period of at least four weeks/28 days. The different results might indicate frequent sick leave episodes after the first event of RTW, which is a known problem for employees with CMDs (Arends et al. 2014). The results point to the relevance of longitudinal studies for evaluating factors associated with RTW. Moreover, the results indicate a need for at-work strategies in RTW interventions for the maintenance of work functioning after the first RTW (Arends et al. 2022).

The association between high psychological job demands and a longer time to first full RTW in our study has been demonstrated in previous studies (Flach et al. 2012; Netterstrøm et al. 2015). The results also align with qualitative research reporting high psychological job demands and inadequate accommodation as barriers to RTW after sick leave due to CMDs (Joosen et al. 2021; Nybergh et al. 2020). Moreover, we found independent associations between high emotional job demands and a longer time to first full RTW, earlier shown in qualitative research (Nybergh et al. 2020). As in our study, Netterstrøm et al. (2015) found low social support at work to be associated with RTW after sick leave due to CMDs. However, in a recent study Sikora et al. (2022) found no such association. Job resources, including high social support and high control, are typically described as a buffer for high psychological job demands on CMDs (Bakker and Demerouti 2017; Karasek and Theorell 1990). However, it has also been suggested that experiencing high psychological job demands over time probably affects employees regardless of resources, and that it is, therefore, important to reduce psychological job demands per se (Bakker and Demerouti 2017; Fagerlind Ståhl et al. 2018; Karasek and Theorell 1990). Moreover, in our study high WHI was independently associated with a longer time to first full RTW. Because WHI is associated with work-related outcomes (Amstad et al. 2011), reducing high psychological job demands may reduce the risk of negative spill-over of job demands into people’s private lives, and benefit the RTW process.

The post hoc analysis showed differences between time intervals for the associations between low job control and high emotional job demands and time to first full RTW. Investigating phase-specific factors is relevant because of the developmental nature of the RTW process (Krause et al. 2001b; Young et al. 2005) and the anticipated impact of RTW policies (Krause et al. 2001a, b) such as time intervals on eligibility for sick leave. Due to the small subgroup (*n* = 35) between > 6 and 12 months, the results of the phase-specific analysis must be interpreted with caution. Although, the differences suggest that future studies should be carried out to determine whether specific factors merit particular attention at specific points in the RTW process.

A strength of the study was the longitudinal design and the follow-up by repeated text messages. Although self-reported sick leave outcomes entail a risk of bias, text messaging has demonstrated high compliance (Axén et al. 2020), and the response rate was 90.2%. Another strength was the use of measures validated in a Scandinavian setting (Berthelsen et al. 2020; Sanne et al. 2005; Wännström et al. 2009). Using single items from the QPS-Nordic is, however, less robust and work-home interference was only investigated with two single items. The recruitment of participants from different PCCs in one Swedish region can help us to extrapolate the results to other Swedish settings.

One of the study’s limitations is the possibility of response and recall bias because of the self-reported data collected retrospectively. Baseline data were collected when the employee was on sick leave. Moreover, the eligibility criteria ‘having to accept employer involvement’, might imply that included participants had a positive perception of their employer and decreased the variance in, and affected the outcome of, fair leadership. Moreover, we do not know exactly when the first full RTW occurred. Because text messages were sent every fourth week, precision was limited. This uncertainty was incorporated into the estimation to reduce the risk of bias. Additionally, missing values preceding the first positive answer were accounted for by widening the censoring interval. Missing values followed by a negative answer were interpreted as a negative answer, ensuring that we did not underestimate the time to the first full RTW. Given the sample size, only the main factors associated with RTW after sick leave due to CMDs were introduced in the adjusted model, the possibility of other confounders needs to be considered in the interpretation of the results. Moreover, because a large majority of the sample were female employees born in Sweden, sub-group analyses based on gender or ethnicity were not possible.

The importance of organisational measures to improve RTW is previously highlighted (cf. Gensby et al. 2019; Joosen et al. 2021; Nielsen et al. 2018). This study implies that workplace accommodations for the employee in rehabilitation after sick leave due to CMDs may well focus on reducing high psychological and emotional job demands, for example by reducing workload, work pace and conflicting/or emotional job demands. Reducing high psychological job demands may also be important to reduce WHI and foster a balanced everyday life for employees (Amstad et al. 2011). In addition, the RTW support offered by primary care and occupational health services should include assessments of the individual’s work and private life to design a sustainable rehabilitation strategy. At the policy level, it is important to acknowledge the adverse effects of high psychological and emotional job demands and high WHI on time to RTW. The estimate for emotional demands in this study is close to one, however, the result corresponds with research showing employees’ experience of emotional demands as a hindrance in RTW (Nybergh et al. 2020) indicating the clinical importance of the results. Moreover, in countries with nearly equal labour-market participation and a gender-segregated labour market, it is important to recognize the possible risks of inequalities for women earlier shown in private life (Eydal et al. 2015) and work domains, such as among care workers (Aronsson et al. 2021; Lidwall et al. 2018). We recommend that future research investigate the clinical importance of emotional demands and the mechanisms of work-home interference in more detail, including the potential differences between social groups. More research is needed to investigate program activities in RTW interventions aiming to reduce high psychological and emotional job demands and high WHI, to further our understanding of the effectiveness of such activities.

## Conclusion

Our study found independent associations between high WHI and a longer time to RTW after sick leave due to CMDs. These have rarely been investigated in earlier research. Moreover, high psychological job demands, high emotional job demands, and low social job support were associated with a longer time to first full RTW. The results underline the need to go beyond work-related factors and to include work-home interference in RTW processes as well.

## Supplementary Information

Below is the link to the electronic supplementary material.

Supplementary file1 (DOCX 29 KB)

## Data Availability

Data is available upon reasonable request and after ethical approval from the Swedish Ethical Review Authority.
