# Supplementary material for: Are psychosocial work factors and work-home interference associated with time to first full return-to-work after sick leave due to common mental disorders?
Source: Int Arch Occup Environ Health. 2023 Mar 25;96(5):747–55. doi: 10.1007/s00420-023-01970-z (PMC10220141; doi:10.1007/s00420-023-01970-z)
Supplement: Supplementary file 1 — Supplementary file1 (DOCX 29 KB) [file 420_2023_1970_MOESM1_ESM.docx]

| Supplementary file 1. Overview of measures for psychosocial work factors and work-home interference | | | | | |
| --- | --- | --- | --- | --- | --- |
|  | **Variable** | **Instrument** | **Subscale/ Single item** | **Questions** | **Score** |
|  | Psychological demands | DSCQ | Scale psychological demands | Does your job require you to work very fast? Does your job require you to work very hard? Does your job require too great a work effort? Do you have sufficient time for all your work tasks? Do conflicting demands often occur in your work? | Four-point scale  (often, sometimes, occasionally, never or hardly) |
|  | Emotional demands | COPSOQ-III | Dimension emotional demands | Does your work put you in emotionally disturbing situations? Do you have to deal with other people’s personal problems as part of your work? Is your work emotionally demanding? | Five-point scale (always, often, sometimes, seldom, never/hardly ever) |
|  | Work-home interference | QPS-Nordic | Single items | (WHI) Do the demands of your work interfere with your home and family life? (HWI) Do the demands of your family or spouse/partner interfere with your work-related activities? | Five-point scale (very  seldom or never; rather seldom; sometimes; rather often; very often or always) |
|  | Job control | DSCQ | Scale decision latitude | Do you have the opportunity to learn new things in your work? Does your work require skills? Does your job require creativity? Does your job require doing the same tasks over and over again? Do you have the possibility to decide for yourself how to carry out your work? Do you have the possibility to decide for yourself what should be done in your work? | Four-point scale (often, sometimes, occasionally, never or hardly) |
|  | Job support | DSCQ | Scale social support | There is a quiet and pleasant atmosphere at my place of work; There is good collegiality at work; My co-workers (colleagues) are there for me (support me); People at work understand that I may have a ‘‘bad’’ day; I get along well with my supervisors; I get along well with my co-workers. | Four-point scale (strongly agree, agree, disagree, strongly disagree) |
|  | Fair leadership | QPS-Nordic | Single item | Does your nearest superior treat the workers fairly and equally? | Five-point scale (very  seldom or never; rather seldom; sometimes; rather often; very often or always) |
| DSCQ=Swedish Demand–Control–Support Questionnaire, COPSOQ-III=Copenhagen Psychosocial Questionnaire III; QPS-Nordic= General Nordic Questionnaire for Psychological and Social Factors at Work; WHI= Work-to-home interference; HWI= Home-to-work interference | | | | | |

**Supplementary file 2.** Descriptive information of psychosocial factors and work-home interference for all participants during the 12-month period, divided into time-intervals of 0-≤6- and >6-12-months, and for participants with no RTW

|  | 12-month period (*n*=162) | | RTW 0-6 months (*n*=96) | | RTW 6-12 months (*n*=35) | | No RTW (*n*=31) | |
| --- | --- | --- | --- | --- | --- | --- | --- | --- |
|  | Mean | SD | Mean | SD | Mean | SD | Mean | SD |
| Psychological demand | 15.0 | 3.3 | 14.4 | 3.2 | 16.0 | 3.6 | 15.8 | 2.8 |
| Job control | 17.4 | 2.6 | 17 | 2.7 | 18.4 | 2.3 | 17.2 | 2.3 |
| Job support | 17.4 | 3.5 | 17.7 | 3.5 | 17.4 | 3.2 | 16.7 | 3.5 |
| Emotional demands | 60.4 | 25.8 | 56.7 | 26.7 | 67.6 | 20.2 | 64.2 | 27.0 |
| Fair leadership | 3.6 | 1.2 | 3.6 | 1.2 | 3.8 | 1.2 | 3.2 | 1.4 |
| WHI | 3.5 | 1 | 3.4 | 0.9 | 3.7 | 1.1 | 3.6 | 0.9 |
| HWI | 2.5 | 1.2 | 2.4 | 1.2 | 2.5 | 1.3 | 2.6 | 1.3 |

WHI= Work-to-home interference; HWI= Home-to-work interference; RTW=return-to-work Scales: Psychological demands (5-20); control and social support (6-24); emotional demands (0-100); leadership (1-5); WHI (1-5). Higher scores indicate higher demands and higher resources.

| Supplementary file 3. Hazard ratio of first full RTW during the 12 months, unadjusted and adjusted models | | | | | | |
| --- | --- | --- | --- | --- | --- | --- |
|  | Unadjusted models | | Adjusted Models | | | |
|  |  |  | Model 1 | Model 2 | Model 3 | |
|  | No. %  (n=162) | HR (95% CI) | HR (95% CI) | HR (95% CI) | HR (95% CI) | |
| Psychological demand | NA | 0.94 (0.89-0.99) | 0.94 (0.89-0.99) | 0.95 (0.90-1.01) | 0.95 (0.90-1.00) | |
| Emotional demands | NA | 0.99 (0.99-1.00) | 0.99 (0.99-1.00) | 0.99 (0.98-1.00) | 0.99 (0.98-1.00) | |
| Job control | NA | 1.03 (0.96-1.10) | 1.02 (0.95-1.10) | 1.05 (0.97-1.13) | 1.04 (0.96-1.12) | |
| Social support | NA | 0.96 (0.92-1.02) | 0.96 (0.91-1.02) | 0.93 (0.88-0.99) | 0.93 (0.88-0.99)^2^ | |
| Fair leadership^1^ |  |  |  |  |  | |
| Ref Fair | 124 (76.5) | 1 | 1 | 1 | 1 | |
| Unfair | 35 (21.6) | 0.92 (0.60-1.42) | 0.90 (0.57-1.41) | 0.95 (0.59-1.54) | 0.94 (0.58-1.53) | |
| WHI^1^ |  |  |  |  |  | |
| Ref. Low | 69 (42.6) | 1 | 1 |  |  | |
| High | 93 (57.4) | 0.78 (0.55-1.11) | 0.78 (0.55-1.11) | 0.62 (0.42-0.91)^2^ | 0.63 (0.43-0.93)^2^ | |
| HWI^1^ |  |  |  |  |  | |
| Ref. Low | 123 (75.9) | 1 | 1 | 1 | 1 | |
| High | 39 (24.07) | 0.93 (0.62-1.39) | 0.78 (0.55-1.11) | 0.80 (0.51-1.27) | 0.79 (0.50-1.25) | |
| HR= Hazard ratio; RTW=return-to-work; WHI=work-to-home interference, HWI=home-to-work interference  HR <1 indicates an increased risk of prolonged RTW.  ^1^Dichotomised: into fair/unfair (unfair=rather seldom and very seldom or never) and high/low WHI (high=rather often and very often or always) | | | Model 1: Adjusted by age and education (primary/secondary education)  Model 2: Additionally adjusted by sick leave  Model 3: Fully adjusted model, by age and education, sick leave, and randomisation (control group) | | |  |

**Supplementary file 4.** Hazard ratio of first full RTW, 0- ≤6- and >6-12-months, and interaction between half years

|  | RTW 0-≤6 months  (n=96) | | RTW >6-12 months^2^  (n=35) | | Interaction between half-years^2^ |  |
| --- | --- | --- | --- | --- | --- | --- |
|  | HR (95% CI) | *p* | HR (95% CI) | *P* | *P* |  |
| Psychological job demands | 0.92 (0.86-0.98) | 0.01 | 1.02 (0.89-1.16) | 0.82 | 0.15 |  |
| Emotional job demands | 0.99 (0.98-1.00) | 0.02 | 1.00 (0.99-1.01) | 0.74 | 0.04 |  |
| Job control | 1.09 (1.00-1.18) | 0.05 | 0.85 (0.73-0.99) | 0.04 | <0.01 |  |
| Social job support | 0.97 (0.92-1.04) | 0.40 | 0.94 (0.84-1.05) | 0.24 | 0.68 |  |
| Fair leadership |  |  |  |  |  |  |
| Ref. Fair | 1 |  | 1 |  |  |  |
| Unfair | 0.96 (0.81-1.15) | 0.70 | 0.81 (0.60-1.08) | 0.16 | 0.36 |  |
| Work-to-home interference |  |  |  |  |  |  |
| Ref. Low | 1 |  | 1 |  |  |  |
| High | 0.69 (0.44-1.07) | 0.09 | 1.08 (0.51-2.31) | 0.84 | 0.24 |  |
| Home-to-work interference |  |  |  |  |  |  |
| Ref. Low | 1 |  | 1 |  |  |  |
| High | 0.93 (0.79-1.11) | 0.43 | 0.98 (0.73-1.32) | 0.90 | 0.80 |  |
| HR= Hazard ratio; WHI= Work-to-home interference; HWI= Home-to-work interference; RTW=return-to-work  HR <1 indicates an increased risk of prolonged RTW  The direction of continuous variables indicate high psychosocial and high emotional job demands and low job control and low social job support. Binary variables are dichotomized into fair/unfair (unfair=rather seldom and very seldom or never) and high/low WHI (high=rather often and very often or always)  ^2^The analysis months is for the difference in the associations between the two time-intervals (0- ≤6- and >6-12-months)  p< 0.05 | | | | | | |
